# Psychosocial and Sexual Aspects of Female Genital Circumcision in a Sample of Kurdish Women in the Kurdistan Region of Iraq

**DOI:** 10.7759/cureus.64881

**Published:** 2024-07-19

**Authors:** Abdulqader Hussein Hamad, Hamdia Mirkhan Ahmed, Aveen Fattah hajimam, Ahmed N Ali, Abdulmalik F Saber

**Affiliations:** 1 Department of Psychiatric and Mental Health Nursing, College of Nursing, Hawler Medical University, Erbil, IRQ; 2 Department of Maternity Nursing, College of Health Sciences, Hawler Medical University, Erbil, IRQ; 3 Department of Maternity Nursing, College of Nursing, Hawler Medical University, Erbil, IRQ

**Keywords:** kurdistan region of iraq, kurdish women, sexual satisfaction, psychosocial impact, female genital circumcision

## Abstract

Background and aim: Female genital circumcision (FGC), a prevalent practice in the Kurdistan Region of Iraq, has significant psychosocial and sexual implications for affected women. Therefore, this study aimed to investigate these impacts among circumcised and non-circumcised Kurdish women.

Method: This comparative study was conducted from January 2 to June 27, 2023, at the Al Mesalla for Human Rights Improvement organization in Erbil, Iraq. Purposive sampling was used to collect data using a comprehensive questionnaire. The questionnaire included demographic information, the Depression, Anxiety, and Stress Scale (DASS-21), the Rosenberg Self-Esteem Scale (RSE), and the New Sexual Satisfaction Scale-Short Form (NSSS-S). Statistical analysis was performed using SPSS version 26 (IBM Corp., Armonk, NY), with frequency and percentage used for categorical variables and mean and standard deviation for quantitative variables. Independent sample t-tests and Chi-square tests were conducted to compare groups. A p-value of less than 0.05 was considered statistically significant.

Results: A total of 772 participants were enrolled in the study, including 382 circumcised and 390 non-circumcised women. The study found significant differences between the two groups in terms of depression, self-esteem, and sexual satisfaction. Circumcised women had higher mean scores for depression (12.19 ± 5.6 vs. 10.68 ± 5.3), lower mean scores for self-esteem (24.4 ± 12.1 vs. 30.3 ± 10.1), and lower mean scores for sexual satisfaction (52.4 ± 24.6 vs. 67.6 ± 20.4) compared to non-circumcised women (all p < 0.001).

Conclusions: The study demonstrated that FGC is associated with higher levels of depression, lower self-esteem, and lower sexual satisfaction among Kurdish women. It is recommended for policymakers and healthcare providers to develop targeted interventions to address the psychosocial and sexual health needs of circumcised women in the Kurdistan Region of Iraq.

## Introduction

Female genital circumcision (FGC), also known as female genital mutilation (FGM) or female genital cutting, is a deeply rooted cultural practice that involves the partial or total removal of external female genitalia for non-medical reasons [1]. This practice affects an estimated 200 million women and girls worldwide, predominantly in 30 countries across Africa, the Middle East, and some Asian countries [2]. In the Kurdistan Region of Iraq, a semi-autonomous region in northern Iraq, FGC continues to be practiced, although its prevalence varies across different communities. According to a study by Shabila [3], the prevalence of FGC in the Kurdistan Region was reported to be approximately 60.6% among women aged 15-49 years. In comparison, other regions of Iraq show lower prevalence rates, such as 8% in the central and southern regions [4]. The WHO classifies FGC into four main types, ranging from partial or total removal of the clitoris to more severe forms involving narrowing of the vaginal opening [5]. FGC is typically performed on young girls between infancy and age 15 and is closely intertwined with cultural beliefs, social norms, and misconceptions about female sexuality and hygiene [6]. Despite international recognition of FGC as a violation of human rights and a form of gender-based violence, it persists due to complex socio-cultural factors.

The implications of FGC extend far beyond the immediate physical consequences. Women who have undergone FGC often face long-term physical, psychological, and sexual health issues [7]. These can include chronic pain, infections, childbirth complications, and various psychological disturbances such as anxiety, depression, and post-traumatic stress disorder [7,8]. Moreover, FGC can significantly impact women's sexual experiences and satisfaction, potentially leading to reduced sexual desire, pain during intercourse, and difficulties achieving orgasm [9]. In the Kurdistan Region of Iraq, FGC practices have been documented, although prevalence rates vary significantly across different areas and communities [3]. The Kurdish culture, with its unique blend of traditional values and modernizing influences, presents a complex backdrop for understanding the persistence of FGC and its effects on women's lives (Yasin et al., 2013). Despite efforts to address FGC through legislation and awareness campaigns, the practice continues to affect a substantial number of Kurdish women [3]. In addition to these challenges, circumcised women often face significant stigma within their communities and broader society. This stigma can manifest in various forms, including social exclusion, discrimination, and negative labeling, which can further exacerbate their psychological distress and social isolation [10-12]. Addressing this stigma is crucial for improving the overall well-being of circumcised women and fostering a more inclusive and supportive environment.

In addition, the psychosocial impact of FGC on Kurdish women is complex and intertwined with cultural norms and personal experiences. Research indicates that women who have undergone FGC often experience higher levels of anxiety and depression compared to non-circumcised women [13]. These mental health challenges can arise from various factors, including the trauma of the procedure itself, ongoing physical discomfort, and the social and cultural pressures surrounding the practice [14]. FGC also greatly affects self-esteem, a crucial aspect of psychological well-being [15]. Women who have undergone the procedure may struggle with body image issues and feelings of incompleteness or inadequacy [16]. Conflicting messages from their communities further complicate these self-perceptions, as FGC is seen as a mark of cultural identity and marriageability but is increasingly recognized as harmful [17].

Another significant concern is the impact of FGC on sexual satisfaction. Research indicates that women who have undergone FGC often report lower levels of sexual satisfaction and more frequent sexual problems compared to non-circumcised women [18]. These issues can range from pain during intercourse to difficulty achieving arousal and orgasm, potentially causing distress and relationship problems [7]. However, it is important to note that the experiences of women who have undergone FGC are not homogeneous [19]. Factors such as the type of FGC performed, the quality of healthcare received, and individual resilience can all influence outcomes [19]. Cultural attitudes towards sexuality and women's bodies also play a significant role in shaping how women perceive and experience their own sexuality after FGC [20].

As we all know, cognitive behavioral therapy (CBT) has been helpful in many cases, such as suicide prevention, anxiety disorders, and depression [21,22]. In our context, CBT can play a crucial role in supporting circumcised women by addressing their mental health challenges and helping them develop healthier coping mechanisms. CBT can assist these women in managing trauma-related symptoms, improving self-esteem, and enhancing their overall psychological well-being [23,24]. By focusing on altering negative thought patterns and behaviors, CBT can provide circumcised women with the tools to overcome the psychological impacts of FGC and lead more fulfilling lives.

Despite the growing body of global research on FGC, there are still significant gaps in understanding its psychosocial and sexual impacts, specifically within the Kurdish population of Iraq. Existing literature tends to focus on African contexts or broader Middle Eastern populations, potentially overlooking the unique cultural nuances and experiences of Kurdish women. Many studies have used qualitative methods or small sample sizes, limiting the generalizability of findings and the ability to make robust comparisons between circumcised and non-circumcised women. Therefore, the present study aims to compare anxiety, stress, depression, self-esteem, and sexual satisfaction among circumcised and non-circumcised women, providing a comprehensive understanding of the psychosocial and sexual impacts of female genital circumcision in the Kurdistan Region of Iraq.

## Materials and methods

Study design, setting, and period

This study was a comparative study conducted at the Al Mesalla for Human Rights Improvement organization in Erbil, Iraq. The purposive sampling method was used to collect data from January 2 to June 27, 2023.

Sample size

To calculate the required sample size for this correlation study, we used a 5% margin of error, a 95% confidence interval, and a population proportion of 50%. Given that the total number of women in the relevant population is approximately 20,000, the required sample size was determined to be 377 cases. However, due to the availability of data, we included 382 cases from the circumcised group and 390 cases from the non-circumcised group.

Inclusion/exclusion

The study's inclusion criteria were women residing in Erbil, aged 15 to under 50, literate, mentally sound, and willing to participate. Both married and unmarried women were included. Exclusion criteria included women who did not complete more than 90% of the questionnaire to ensure data integrity and women with severe cognitive impairments that could affect their ability to provide informed consent and reliable responses.

Study tools and data collection

The questionnaire was divided into four main parts. The first part gathered demographic data, including age group, level of education, level of education of husband, occupation, occupation of husband, residential area, economic status, and house ownership. The second part was the Depression, Anxiety, and Stress Scale (DASS-21) questionnaire to assess depression, stress, and anxiety. The third part included the Rosenberg Self-Esteem Scale (RSE), which contained 10 items to measure self-esteem. The fourth part focused on sexual satisfaction using the New Sexual Satisfaction Scale-Short Form (NSSS-S), which contained 20 items to assess sexual satisfaction. The questionnaire was translated from English to Kurdish using a forward-backward method to ensure accuracy, and the translation was verified by a psychiatrist in the field. Data were collected by distributing questionnaires to participants who met the inclusion criteria. The administration process involved explaining the study's purpose and procedures to participants, ensuring their understanding and voluntary participation. Each participant was allotted a total of 10-15 minutes to complete the questionnaire, with trained researchers available to assist if needed. The completed questionnaires were then collected for data analysis.

Pilot study

The study questionnaires were tested in an initial study with 20 participants for each tool from the general population on October 10, 2022. The internal consistency and reliability of the items were assessed over a period of one month, with tests retaken after this period. The internal consistency of the items was calculated using Cronbach's alpha [25]. For the DASS-21, the overall Cronbach's alpha was 0.82, indicating very good reliability. For the RSE, the Cronbach's alpha was 0.86, again demonstrating very good reliability. For the NSSS-S, the Cronbach's alpha was 0.85, indicating very good internal consistency. These results demonstrate that all tools have acceptable internal consistency and reliability. The data from this initial study were excluded from the final analysis.

Measures

Sociodemographic Characteristics

The demographic data included various variables measured during the study, such as age group, level of education, level of education of husband, occupation, occupation of husband, residential area, economic status, and house ownership.

Depression, Anxiety, and Stress Scale - 21 Items

The second part of the questionnaire included the Depression, Anxiety, and Stress Scale - 21 Items (DASS-21) [26]. This scale was designed to measure levels of depression, anxiety, and stress. It consisted of 21 statements, each rated on a four-point Likert scale (0: did not apply to me at all, 1: applied to me to some degree or some of the time, 2: applied to me to a considerable degree or a good part of the time, 3: applied to me very much or most of the time). Scores for depression, anxiety, and stress are calculated by summing the scores for the relevant items and multiplying by 2 to calculate the final score. The recommended cut-off scores for conventional severity labels are as follows: Depression: Normal (0-9), Mild (10-13), Moderate (14-20), Severe (21-27), Extremely Severe (28+); Anxiety: Normal (0-7), Mild (8-9), Moderate (10-14), Severe (15-19), Extremely Severe (20+); Stress: Normal (0-14), Mild (15-18), Moderate (19-25), Severe (26-33), Extremely Severe (34+). The total scores across the three subscales indicated the severity of symptoms, with higher scores indicating greater severity. The internal consistency of the DASS-21 was assessed using Cronbach's alpha [25], which resulted in a reliability score of 0.82, indicating acceptable internal consistency.

Rosenberg Self-Esteem Scale

The third part of the questionnaire included the RSE scale [27]. This scale was designed to measure self-esteem. It consisted of 10 statements, each rated on a four-point Likert scale (1: strongly agree, 2: agree, 3: disagree, 4: strongly disagree). The scale can be scored by totaling the individual 4-point items after reverse-scoring the negatively worded items. Low self-esteem responses are “disagree” or “strongly disagree” on items 1, 3, 4, 7, 10, and “strongly agree” or “agree” on items 2, 5, 6, 8, 9. The total score indicated the level of self-esteem, with higher scores representing higher self-esteem. The internal consistency of the RSE was assessed using Cronbach's alpha [25], which resulted in a reliability score of 0.86, indicating very good internal consistency.

New Sexual Satisfaction Scale-Short Form

The fourth part of the questionnaire included the NSSS-S [28]. This scale was designed to measure sexual satisfaction. It consisted of 20 statements, each rated on a five-point Likert scale (1: not at all satisfied, 2: a little satisfied, 3: moderately satisfied, 4: very satisfied, 5: extremely satisfied). The scale is divided into two subscales: the Ego-Centered Subscale (items 1-10) and the partner- and Activity-Centered Subscale (items 11-20). Scores are computed by summing the related items, with higher scores representing higher levels of sexual satisfaction. The total scores indicated the level of sexual satisfaction, with higher scores representing higher satisfaction. The internal consistency of the NSSS-S was assessed using Cronbach's alpha [25], which resulted in a reliability score of 0.85, indicating very good internal consistency.

Ethical Approval and Inform Consent

This study followed the Institutional Research Ethics Board and the Declaration of Helsinki guidelines. The Scientific and Ethical Committee of the College of Nursing at Hawler Medical University, as well as the Health Directorate of Erbil Governorate, approved the study's ethics (ethical code number: 130, date: December 25, 2022). We had a written agreement with the Al Mesalla for Human Rights Improvement organization outlining our procedures. For participants under 18, written consent was obtained from both the participants and their parents, including their signatures. For participants above 18, both oral and written informed consent were obtained, ensuring all participants understood the study and voluntarily agreed to participate. The informed consent process was explained in detail, including the purpose of the study, procedures involved, potential risks and benefits, and the voluntary nature of participation. Participants were assured of their right to withdraw from the study at any time without any consequences.

Statistical Analysis

The data were summarized and reported with frequency and percentage for qualitative variables. Quantitative variables with a normal distribution were presented with means and standard deviations. Independent sample t-tests and Chi-square tests were used to compare demographic data for continuous and categorical variables, respectively. Given that our data is not normally distributed, we have used non-parametric tests such as Chi-square to compare the groups. Data analysis was performed using SPSS version 26 (IBM Corp., Armonk, NY), with significance levels considered at P < 0.05.

## Results

Demographic characteristics

A total of 772 responses were analyzed, with all participants successfully completing the questionnaire. The mean age of circumcised women was 33.12 ± 9.53 years, while for non-circumcised women it was 29.55 ± 9.30 years. A significant difference was observed in the age group distribution, with 65.4% of circumcised women being above 38 years compared to 34.6% of non-circumcised women (P=0.001). Educational levels also showed significant differences, with 61.7% of illiterate women being circumcised compared to 38.3% non-circumcised (P=0.04). The residential area distribution indicated that 56.7% of women in rural areas were circumcised compared to 43.3% non-circumcised, while in urban areas, 44.6% of women were circumcised compared to 55.4% non-circumcised (P=0.001). No significant differences were found in the economic status, house ownership, or husband's occupation between the two groups (Table 1).

**Table 1:** Demographic characteristics of circumcised and non-circumcised women Circumcised women numbered 382, and non-circumcised women numbered 390, making a total of 772 participants. Chi-square and independent sample t-tests were used for data analysis. Significance was set at P < 0.05.

Variable	Type of variable	Group classification		P-value
		Circumcised women no. (%)	Non-circumcised women no. (%)	
Age group	<19	7(21.9)	25(78.1)	0.001
	19–28	142(45.4)	171(54.6)	
	29–38	108(45.8)	128(54.2)	
	>38	125(65.4)	66(34.6)	
	Mean ± SD	33.12 ± 9.53	29.55 ± 9.30	
Level of education	Illiterate	66(61.7)	41(38.3)	0.04
	Read and write	51(49.0)	53(51.0)	
	Primary school	86(51.8)	80(48.2)	
	Secondary school	76(43.4)	99(56.6)	
	Institute and above	103(46.8)	117(53.2)	
Level of education of husband	Illiterate	38(61.3)	24(38.7)	0.20
	Read and write	50(54.3)	42(45.7)	
	Primary school	77(49.7)	78(50.3)	
	Secondary school	98(48.5)	104(51.5)	
	Institute and above	119(45.6)	142(54.4)	
Occupation	Housewife	272(51.6)	255(48.4)	0.08
	Employee	110(44.9)	135(55.1)	
Occupation of husband	Employee	157(49.4)	161(50.6)	0.84
	Unemployed	207(49.2)	214(50.8)	
	Retired	18(54.5)	15(45.5)	
Residential area	Rural	177(56.7)	135(43.3)	0.001
	Urban	205(44.6)	255(55.4)	
Economic status	Sufficient	73(52.1)	67(47.9)	0.43
	Somehow sufficient	280(49.6)	284(50.4)	
	Insufficient	29(42.6)	39(57.4)	
House ownership	Owned	293(51.1)	280(48.9)	0.14
	Rented	89(44.7)	110(55.3)	

Psychological and sexual health outcomes

The mean depression score was higher among circumcised women (12.19 ± 5.6) compared to non-circumcised women (10.68 ± 5.3). The mean anxiety score for circumcised women was 8.07 ± 4.24, slightly higher than for non-circumcised women (7.44 ± 4.22). For stress, the mean score was 14.88 ± 7.03 for circumcised women and 13.54 ± 6.98 for non-circumcised women. The mean self-esteem score was lower among circumcised women (24.4 ± 12.1) compared to non-circumcised women (30.3 ± 10.1). Sexual satisfaction scores indicated a mean of 52.4 ± 24.6 for circumcised women and 67.6 ± 20.4 for non-circumcised women (Table 2).

**Table 2:** Comparison of psychological and sexual satisfaction variables between circumcised and non-circumcised women Circumcised women numbered 382, and non-circumcised women numbered 390, making a total of 772 participants. Chi-square was used for data analysis. Significance was set at P < 0.05.

Variable	Category	Group classification		P-value
		Circumcised women no. (%)	Non-circumcised women no. (%)	
Depression	Normal	100 (38.6%)	159 (61.4%)	0.001
	Mild	136 (56.0%)	107 (44.0%)	
	Moderate	123 (53.2%)	108 (46.8%)	
	Severe	23 (60.5%)	16 (39.5%)	
	Mean ± SD	12.19 ± 5.6	10.68 ± 5.3	
Anxiety	Normal	150 (44.6%)	186 (55.4%)	0.13
	Mild	103 (53.1%)	91 (46.9%)	
	Moderate	102 (53.4%)	89 (46.6%)	
	Severe	27 (52.9%)	24 (47.1%)	
	Mean ± SD	8.07 ± 4.24	7.44 ± 4.22	
Stress	Normal	150 (43.9%)	192 (56.1%)	0.06
	Mild	113 (53.8%)	97 (46.2%)	
	Moderate	99 (53.5%)	86 (46.5%)	
	Severe	20 (57.1%)	15 (42.9%)	
	Mean ± SD	14.88 ± 7.03	13.54 ± 6.98	
Self-esteem	Strongly disagree	128 (78.0%)	36 (22.0%)	0.001
	Disagree	61 (43.6%)	79 (56.4%)	
	Agree	90 (47.6%)	99 (52.4%)	
	Strongly agree	103 (36.9%)	176 (63.1%)	
	Mean ± SD	24.4 ± 12.1	30.3 ± 10.1	
Sexual satisfaction	Not at all satisfied	92 (93.9%)	6 (6.1%)	0.001
	A little satisfied	91 (48.1%)	98 (51.9%)	
	Moderately satisfied	90 (56.6%)	69 (43.4%)	
	Very satisfied	87 (33.0%)	177 (67.0%)	
	Extremely satisfied	22 (35.5%)	40 (64.5%)	
	Mean ± SD	52.4 ±24.6	67.6± 20.4	

## Discussion

The present study aimed to compare anxiety, stress, depression, self-esteem, and sexual satisfaction among circumcised and non-circumcised women. Understanding the multifaceted impacts of FGC is crucial for developing effective interventions and support systems for affected women. Overall, the results revealed significant demographic differences, with circumcised women being generally older and more likely to be from rural areas. The psychological health findings indicated higher levels of depression, anxiety, and stress among circumcised women. Additionally, circumcised women had lower self-esteem and reported lower levels of sexual satisfaction compared to their non-circumcised counterparts.

The FGC is a pressing public health and human rights concern that profoundly impacts millions of women worldwide [1]. It results in significant physical, psychological, and sexual health repercussions [7]. Despite global efforts to eliminate this practice, it persists in various communities, including certain regions within the Kurdistan Region of Iraq, due to deeply ingrained cultural beliefs and social norms [29]. While the long-term effects of FGC on women's well-being have been extensively studied, it is crucial to conduct context-specific research to comprehend the unique challenges faced by different populations. Therefore, our research focuses on shedding light on the specific consequences of FGC among Kurdish women in the Kurdistan Region of Iraq.

The demographic analysis revealed clear differences between circumcised and non-circumcised women. Circumcised women tended to be older and more likely to come from rural areas. This finding is consistent with previous research that has shown higher rates of FGC in rural areas and among older generations [30]. The urban-rural divide can be attributed to factors such as limited access to education, a stronger adherence to traditional practices, and less exposure to campaigns against FGC in rural settings. However, it is important to note that FGC is not uniformly practiced in all rural areas, and variations exist depending on local customs, religious interpretations, and community leadership [19]. Another significant difference between circumcised and non-circumcised women is the level of education. A higher proportion of circumcised women were found to be illiterate, which is consistent with findings from other studies [5,31]. Education is recognized as a protective factor against FGC as it empowers women to challenge harmful traditions and make informed decisions about their health [31]. However, the relationship between education and FGC is complex, and studies have found that education alone may not be sufficient to eliminate the practice, especially in communities where FGC is deeply rooted in their cultural identity [5,31].

The study's findings on psychological health showed that circumcised women experienced higher levels of depression, anxiety, and stress compared to non-circumcised women. This aligns with a growing body of evidence linking FGC to negative mental health outcomes [13]. The psychological impact of FGC can be attributed to factors such as the traumatic nature of the procedure, chronic pain, and complications arising from the circumcision [7]. The social and cultural context in which FGC occurs also contributes to psychological distress as women navigate conflicting messages about their bodies and cultural expectations [20]. Potential cultural factors in the Kurdistan Region that exacerbate this distress include the deep-rooted belief in FGC as a rite of passage and a marker of cultural identity [7]. Additionally, the pressure to conform to traditional gender roles and the stigma associated with rejecting FGC can lead to significant emotional turmoil [7]. These cultural expectations, coupled with increasing awareness about the harmful effects of FGC, create a complex environment that intensifies the psychological impact on circumcised women. However, it is important to note that the relationship between FGC and mental health is complex and can be influenced by various mediating factors. Studies have found that social support, cultural beliefs, and individual coping mechanisms can moderate the psychological impact of FGC [32,33]. The higher levels of psychological distress observed in this study may be particularly significant in the specific cultural context of the Kurdistan Region, where increasing awareness about the harmful effects of FGC may lead to internal conflicts for circumcised women.

According to the study's findings on self-esteem, circumcised women generally reported lower self-esteem compared to non-circumcised women. This finding aligns with previous research that has documented the negative impact of FGC on women's self-perception and self-worth [16]. The lower self-esteem among circumcised women may be attributed to various factors, including societal stigma, feelings of physical inadequacy, and the internalization of negative messages about their bodies.

The study shows that circumcised women report significantly lower levels of sexual satisfaction compared to non-circumcised women. This highlights the negative impact of FGC on sexual health and well-being. Similar findings have been documented in several studies, which have consistently shown reduced sexual function, pleasure, and satisfaction in women who have undergone FGC [7,18]. Various factors contribute to the decrease in sexual satisfaction, such as physical damage to sensitive genital tissues, chronic pain, and psychological trauma associated with the procedure. However, it is important to acknowledge that sexuality is a complex aspect of human experience influenced by cultural, social, and individual factors [34]. Some studies have found that despite the physical consequences of FGC, some women still report satisfactory sexual experiences, suggesting the role of psychological and relational factors in sexual satisfaction [5,35]. The lower sexual satisfaction observed in this study may be due to both the physical consequences of FGC and changing cultural attitudes towards sexuality and women's rights in the Kurdistan Region.

Addressing the sexual health needs of circumcised women requires a comprehensive approach involving medical interventions, psychological support, and sexual education. Healthcare providers should receive training to deliver sensitive and culturally appropriate care to women who have undergone FGC, including counseling on sexual health and, if appropriate, reconstructive surgeries. Moreover, community-based interventions that encourage open dialogue about sexuality and challenge harmful myths surrounding FGC can contribute to improving the sexual well-being of affected women. Furthermore, future research should focus on developing and evaluating comprehensive intervention programs that integrate medical, psychological, and educational support for circumcised women. Additionally, studies should explore the effectiveness of community-based initiatives aimed at fostering open dialogue and challenging cultural myths about FGC to enhance the sexual well-being of affected women.

Limitation of the study

A key limitation of the study is that its findings are specific to the Al Mesalla for Human Rights Improvement organization in Erbil and may not be generalizable to other regions of Iraq or other populations. Additionally, the use of purposive sampling may introduce bias, which may affect the study's overall validity.

## Conclusions

The study demonstrated that FGC is associated with higher levels of depression, lower self-esteem, and lower sexual satisfaction among Kurdish women. Policymakers and healthcare providers should develop targeted interventions to address these psychosocial and sexual health challenges. Educational programs are essential to raising awareness about the negative impacts of FGC. Support services, including mental health counseling, should be made accessible to affected women. These efforts could significantly improve the overall well-being and quality of life for circumcised women in the Kurdistan Region of Iraq. Future research should explore the long-term psychological and sexual health outcomes of FGC, investigate the effectiveness of different intervention strategies, and examine the role of cultural factors in moderating these impacts. Additionally, studies focusing on younger cohorts and diverse regions within Iraq could provide a more comprehensive understanding of the FGC's effects.
